# Dr. M’Dowell and His Pretensions to the Cure of Consumption

**Published:** 1844-10

**Authors:** 


### DR. M'DOWELL AND HIS PRETENSIONS TO THE CURE OF CONSUMPTION.

An article from the Louisville Journal, written by Dr. M'Dowell in reply to our late notice of his book, is to be served up, we understand, to the readers of this Journal. We declined its publication because it appeared to us to be no better than the advertisement, the insertion of which in our January number excited among our friends such general surprise. We shall not waste much time upon this pamphlet, but a few words of explanation would seem to be called for, and we may be tempted, before we quit the subject, to mention some of the circumstances to which is owing the universal distrust felt by Dr. M'Dowell's professional brethren of this city in the reality of his cures.

Dr. M'Dowell alludes, triumphantly, to the fact that we gave an editorial notice of his cases at the end of his article in our January number. He deems it worthy of several notes of admiration, and, for once, we think he is right. It was, indeed, a great oversight in us, for which our present controversy with him is but a just punishment. The manner in which it happened was this:—Dr. M'Dowell called at our residence with his communication, at the end of which we found it stated that "a list of the names of 84 cases" was left with the editors of the Journal. We declined taking charge of his list, but suggested that it might be left at the Journal office, and, with a good nature which we now feel to have been unpardonable, made the editorial statement to which he refers, adopting almost the very language of the communication. We thus saved him the necessity of advertising his own cases; and he now turns upon us and taunts us with the kind act. The punishment, we repeat, is just.

Dr. M'Dowell accuses us of "injustice in charging him with claiming originality in regarding consumption as curable in all its stages." He adds, "I have advanced no such claim." Now, in his book, (page 57) he says, "the most eminent pathologists concur in the opinion that pulmonary consumption is curable even in the last stage; but, singularly enough, they are equally concurrent that it is curable in that stage *only*." If this is not a claim of originality—a claim to be the first among the "great pathologists" to teach that it is curable in other stages than the last, we desire to know what the language means. Nor does it help the matter at all, that, a few pages further on, he cites a case of pththisis from Stokes, as cured in the first stage, which Stokes does not give as a case of phthisis; for if he had really adduced such testimony against his first proposition, it would only have confirmed our statement in the review, that he sets up claims in one paragraph to be nullified by evidence brought forward in another. Even in his very brief reply he has not been able to avoid such absurdities, for while at the beginning he modestly disclaims all originality, we find him, before he reaches the close, ranking himself with Harvey and Jenner, and that class of medical worthies, whose discoveries, he thinks, hardly met so fierce a storm of opposition as his have had to encounter. But we pass from these venial offences.

Dr. M'Dowell claims to cure consumption in all its stages. A few years since he professed to have effected a radical cure in a young lady of this city. Sometime subsequently the lady died of another disease, and Dr. M'D. carried her lungs to a club of medical gentlemen in the city, to demonstrate the cure. They were carefully dissected before the club, but not a vistige of tubercular disease could be detected—not the slightest trace of an ulcer cicatrised. The club remembered that cicatrices had been found in the lungs many years after the healing of an ulcer, and the president asked Dr. M'D. how he accounted for their absence in his case? "*Cured so completely*," was his answer, "*that no cicatrix remained.*" A very intelligent member replied, that he "could as easily believe that *a pig's stump tail would grow out again;*" and our readers will agree with him, that one event is just about as probable as the other.

Another case, and equally illustrative of Dr. McD's skill in diagnosis. A lady, far gone in consumption, became his patient after a number of physicians had pronounced her case hopeless. He assured her husband, a short time before she died, that he had succeeded in *removing the tubercles* from her lungs. Not a tubercle was to be disclosed by the *post mortem* examination; this was his prediction. What were the facts, as witnessed by several of the most eminent physicians in the city? One lobe of the lungs was occupied by *a tuberculous mass that would have filled a man's two hands*—was but little else than a mass of softened tuberculous matter; and the opposite lobe contained two or three tubercles as large as a walnut! Of course, Dr. McD. looked astonished.

Our readers can understand, after this, why it is that Dr. McDowel's medical neighbors not only do not believe in his reported cures of consumption, but even seriously doubt his ability to make out the diagnosis of the disease. Not by a few "in chimney corners," as he insinuates, but by the whole body of scientific physicians in Louisville, where his practice has been tested, his pretensions are utterly repudiated and scouted. Many of these practitioners are our common friends; some are personally friendly to him who sustain no such relations to us. Can he get from any of them a certificate, that he ever cured a case of tubercular consumption? Let him produce such a certificate from a physician who is known to understand the physical signs of diseases of the lungs, and afterwards he may come before the profession with some chances of being believed. Y.

